# A Case of Sudden Death: Subarachnoid Hemorrhage, Pheochromocytoma, Berry Aneurysm

**DOI:** 10.1155/2019/2905078

**Published:** 2019-06-10

**Authors:** Ana B. Arevalo, Jacxelyn Moran, Stephen Zink, Binny Khandakar

**Affiliations:** ^1^Department of Internal Medicine, Icahn School of Medicine at Mount Sinai West-St. Luke's, New York, NY 10019, USA; ^2^Department of Diagnostic Radiology, Icahn School of Medicine at Mount Sinai West-St. Luke's, New York, NY 10019, USA; ^3^Department of Diagnostic Radiology, Mount Sinai Health System, New York, NY 10019, USA; ^4^Department of Pathology, Mount Sinai Health System/Icahn School of Medicine at Mount Sinai West-St. Luke's, New York, NY 10019, USA

## Abstract

Subarachnoid hemorrhage is a medical emergency. Berry aneurysm rupture is the second most common cause following trauma. Diagnosis is often challenging. Neurogenic heart syndrome often complicates subarachnoid hemorrhage. A concomitant pheochromocytoma can be deadly causing sudden cardiac arrhythmia. Here, we describe a case of subarachnoid hemorrhage with concomitant incidental pheochromocytoma in a relatively young female who died suddenly, diagnosed during autopsy. A 57-year-old Hispanic woman with past medical history of asthma, prediabetes, and uncontrolled hypertension collapsed unexpectedly. She initially had ventricular tachycardia, followed by pulseless electrical activity and finally asystole without response to resuscitation. In the emergency department she was on epinephrine, calcium, naloxone, and tPA with suspected thrombotic stroke. Despite measures, she was pronounced dead. Autopsy revealed subarachnoid hemorrhage due to a ruptured berry aneurysm. Additionally, pheochromocytoma was detected in the right adrenal gland. Subarachnoid hemorrhage has a grave prognosis by itself. This case describes the uncommon detection of pheochromocytoma in the setting of subarachnoid hemorrhage.

## 1. Introduction

Sudden death is defined as death occurring rapidly and unexpectedly, usually from a cardiac cause such as dysrhythmia or myocardial infarction, but also from any cause leading to rapid mortality, for example, pulmonary thromboembolism, stroke, ruptured aneurysm, or aortic dissection [1]. The following case report describes the clinical, radiological, and autopsy findings of a previously “otherwise healthy” 57-year-old Hispanic woman who died suddenly in the emergency department. Postmortem evaluation revealed subarachnoid hemorrhage (SAH) due to a rupture of a berry aneurysm likely driven by a paroxysm of hypertension in the setting of an undiagnosed and untreated pheochromocytoma.

## 2. Case Report

### 2.1. Initial Presentation

A bystander found the unresponsive patient in the field and CPR (cardiopulmonary resuscitation) was started approximately 10 minutes after collapse. She initially had ventricular tachycardia; however, she did not regain pulses and had pulseless electrical activity before asystole despite the use of a defibrillator. During this time, she received epinephrine and was intubated prior to arriving in the emergency department. In the emergency room, the patient remained unresponsive, without pulses and without spontaneous breathing. Physical examination showed fixed dilated pupils. CPR was continued with further administration of epinephrine, calcium, naloxone, and tPA. The patient did not regain pulses. Bilateral needle thoracostomy was also attempted. Without return of spontaneous circulation, she was finally pronounced dead. The family requested for autopsy to find out the cause of death.

### 2.2. Autopsy: Gross and Microscopy

**Figure 1:**
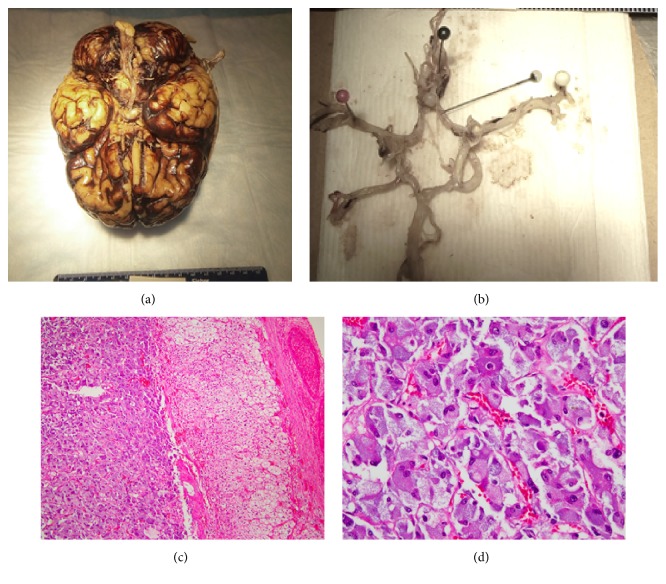
(a) Gross photograph: brain, subarachnoid hemorrhage. (b) Gross photograph: circle of Willis, ruptured berry aneurysm. (c) Hematoxylin & Eosin Stain (100x): adrenal gland with tumor in the medulla showing nesting pattern. (d) Hematoxylin & Eosin Stain (400x): tumor cells from pheochromocytoma showing large polygonal tumor cells with amphophilic cytoplasm.

A “no restrictions” autopsy was performed. Externally no signs of trauma were observed. Gross examination of organs systems revealed an enlarged thyroid gland (weight: 75 grams, normal: 10–30 grams) with an expanded multinodular right lobe, a cystic and solid mass of the right adrenal gland (4.6 x 3.5 x 2.5 cm; weight: 71 grams, normal: 4-6 grams), moderate-to-severe stenosis of all three coronary vessels, and a scar-like area involving posterior wall of left ventricle. Examination of brain showed subarachnoid hemorrhage (Figure 1(a)). Dissection of the intracranial vasculature revealed a ruptured berry aneurysm at the junction of right cerebral and anterior communicating arteries (Figure 1(b)). On microscopy, sections from the adrenal mass showed a tumor arising in the adrenal medulla, composed of tumor cells arranged in a Zellballen pattern. Individual cells were large polygonal cells with abundant amphophilic to eosinophilic cytoplasm, displaying mild-to-moderate nuclear-cytologic atypia and minimal mitosis. Based on morphology, the adrenal tumor was diagnosed as pheochromocytoma (Figures 1(c) and 1(d)). Other findings included myocardial hypertrophy and fibrosis indicative of healed infarcts, moderate-to-severe atherosclerosis of coronary arteries with focal dystrophic calcification, adenomatoid nodule of thyroid gland, focal minimal interstitial chronic inflammation of kidneys, mild chronic interstitial inflammation and smoker's type pigment deposition involving bilateral lungs, and internal carotid artery with moderate stenosis and calcification. The final cause of death was subarachnoid hemorrhage due to rupture of an intracranial berry aneurysm in the setting of a right adrenal pheochromocytoma, chronic ischemic heart disease, and three-vessel moderate-to-severe coronary stenosis.

### 2.3. Longitudinal Medical History

**Figure 2:**
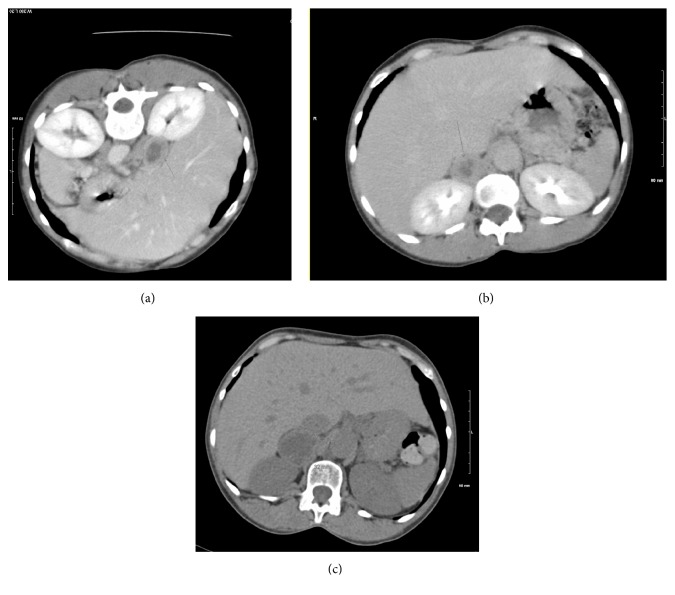
(a-b) CT abdomen and pelvis with contrast in 2006 demonstrating a large right adrenal mass with a hypodense center (a) venous phase and (b) delayed imaging. (c) Noncontrast CT abdomen pelvis in 2013 showing interval increase in size of the right adrenal mass to 3.7 x 3.2 cm from 2.3 x 1.9 cm.

A thorough evaluation of her past medical history was performed to supplement and correlate the autopsy findings. Her prior medical history included asthma, prediabetes, uncontrolled hypertension, tobacco use, chronic lower back pain with scoliosis, multinodular goitre, osteoporosis, and depression. She was known to the emergency department for intermittent bouts of abdominal pain, vomiting, and nausea. Prior work-up by CT abdomen and pelvis imaging in 2006 demonstrated an enhancing right adrenal mass with a hypodense center, likely necrosis, without demonstration of washout (Figures 2(a) and 2(b)). Despite recommendations for follow-up MRI imaging, no further intervention was done. The mass had an interval increase from 2007 to 2013 from 2.8 x 2.2 cm to 3.7 x 3.2 cm (Figure 2(c)). Her urinalysis as an outpatient was significant for concentrated urine, ketones, microscopic hematuria, and proteinuria. Her labs in 2011 were unremarkable with normal comprehensive metabolic panel, lipid panel, TSH, and CBC.

### 2.4. Clinicopathologic Correlation

History of poorly controlled hypertension and adrenal mass on imaging, pheochromocytoma, was a differential in retrospect. Further digging into the history revealed ongoing symptoms of palpitations, significant weight loss with a BMI of 16.5 kg/m2, insomnia, intermittent shortness of breath, anorexia, depression, and anxiety. She denied bowel changes and tremor. However, urinary metanephrines or normetanephrines were unavailable in the record.

## 3. Discussion

Association of subarachnoid hemorrhage (SAH) and pheochromocytoma has rarely been described in literature; the first case report is from 1952 [2]. To the best of our knowledge, this is the third case of this nature. Clark and colleagues described the unexpected demise of a hypertensive patient due to cardiac arrest, who was later found to have pheochromocytoma [3]. Our unique patient presented with SAH due to a ruptured intracranial aneurysm in the setting of uncontrolled hypertension and an undiagnosed functional pheochromocytoma which was discovered during autopsy. The current case focuses on the interplay of pheochromocytoma on the pathophysiology of intracranial saccular aneurysm formation and risk of rupture and highlights the clinical implications of this distinctive patient scenario.

Worldwide, almost 500,000 individuals suffer from aneurysmal subarachnoid hemorrhage, with almost two-thirds of cases occurring in low- and middle-income countries. Approximately 80% of spontaneous SAHs are associated with aneurysmal rupture [4, 5]. Risk of subarachnoid hemorrhage in hypertensive crisis is significantly magnified in patients with an intracranial aneurysm (1-2% incidence in the general population). The most common type of intracranial aneurysm, as well as the leading cause of nontraumatic aneurysmal subarachnoid hemorrhage (SAH), is the saccular intracranial aneurysm [5]. The risks for developing saccular aneurysm and subsequent SAH are multifactorial, including both modifiable and nonmodifiable risk factors. Prospective population-based studies have shown smoking, untreated hypertension, and female gender as important risk factors. Although smoking influences both the formation and rupture of aneurysms, other risk factors such as gender and hypertension only affect the risk of aneurysm formation. In experimental animal studies, formation of cerebral artery aneurysms was induced by hypertension through disrupted collagen synthesis [5, 6]. Pathophysiology involves an initial disruption of internal elastic laminae and death of smooth muscle cells in the tunica media, followed by outpouching of the arterial wall with histiocyte-rich inflammation. Autosomal inherited diseases, including polycystic kidney disease, Ehlers-Danlos type IV, and fibromuscular dysplasia, have also been associated with intracranial aneurysm. Although a positive family history of SAH or saccular aneurysm is a risk factor for SAH, the degree of genetic penetration varies, as evident by findings from a twin-based study that did not show significant genetic contribution [7, 8]. Population-based studies have revealed association of single nucleotide polymorphism with saccular aneurysm and several genetic loci have been reported. Genetic polymorphism related to cerebral artery wall structure might increase the risk of aneurysm formation and rupture; however, this risk is less than factors such as smoking, hypertension, and female gender [9].

Pheochromocytomas are rare chromaffin cell tumors, arising from the adrenal medulla, producing epinephrine, dopamine, and norepinephrine with varied symptomatology. It has a prevalence of 0.2-0.6% in patients with hypertension and 3-7% in those with adrenal incidentalomas [10]. The symptoms range from asymptomatic clinical presentation to poorly controlled hypertension, anxiety, sleep disturbances, weight loss, palpitations, headache, and even sudden cardiac arrest [4, 10, 11]. Diagnosis and treatment are often delayed due to vague and overlapping clinical picture.

In retrospect, our patient had both modifiable and nonmodifiable risk factors for the development and rupture of saccular aneurysm. Although a lack of microscopic examination of the aneurysmal wall may be a limitation in terms of morphologic examination of the aneurysm, the patient's female sex, tobacco use, and history of uncontrolled hypertension undeniably contributed to the development and exacerbation of her intracranial saccular aneurysm as well as to its eventual rupture. Each risk factor, either individually or in combination, might have impacted the creation and or instability of the saccular aneurysm, which could have been there for a long time, hitherto undiagnosed until it ruptured. Catecholamine surges from her undiagnosed pheochromocytoma undoubtedly predisposed her to paroxysms of hypertension, leading to repeated arterial wall trauma, and possibly even genesis, of her intracranial berry aneurysm. The exact pathophysiology of the intracranial aneurysm and SAH in this patient would remain a mystery, but was probably influenced by the patient's sex, hypertension, behavioral risk factor, and unique neurohormonal state due to the pheochromocytoma.

The current patient presented with the pathologic sequelae of ventricular tachycardia and cardiac arrest in the setting of SAH and unidentified ‘functional' pheochromocytoma. Patients with pheochromocytoma and hypertension have a 14-fold increased risk of cardiovascular events such as heart attack or stroke, with severe hypertension occurring as often as once weekly in about 75% of patients [12]. Similar to a pheochromocytoma, SAH causes an imbalance in the autonomic nervous system with a subsequent increased sympathetic output and an elevation of circulating catecholamines [13]. This tilt in normal neural homeostasis can lead to neurogenic heart syndrome, a condition where overstimulation of the sympathetic nervous system leads to cardiac arrhythmias, ultimately leading to cardiac arrest [14]. Hence the exaggerated circulating levels of catecholamines and the use of sympathomimetic drugs as part of cardiac arrest protocol may have accounted for arrhythmia causing death in this patient.

A diagnosis of acute ‘pheochromocytoma crisis' should remain on the differential in patients with acute cardiovascular shock [15]. A quick diagnosis can be done by rapid imaging such as contrast-enhanced CT [16, 17]. Although an optimal method of treating the acute presentation of a functional pheochromocytoma has not been established, treatment of acute ‘pheochromocytoma crisis' includes initial blood pressure and heart rate control with alpha-adrenergic receptor blockers, with optional rhythm control via beta-blockers or calcium channel blockers, followed by surgical resection within 10-14 days [16]. Like functional pheochromocytomas, aneurysmal SAH can also present as a medical emergency that is frequently misdiagnosed, yielding dreadful immediate and delayed consequences such as hydrocephalus, seizures, cerebral ischemia, herniations, hyponatremia, cardiac abnormalities, and respiratory depression [17]. The best diagnostic study to exclude an SAH is a noncontrast CT head; if positive suspicion for aneurysm exists, then this should be followed by a contrast-enhanced CT angiography of the head and neck [18]. Between the time of SAH symptom onset and aneurysm rupture, blood pressure should be controlled with a titratable agent balancing the risk of stroke and rebleeding and maintaining cerebral perfusion pressure. Vascular intervention, clipping, or coiling of the ruptured aneurysm should be performed as early as feasible, to reduce rebleeding [17, 19].

In order to avoid deadly scenarios, such as the one presented in this case, certain preventative measures may be implemented in clinical setting. Pheochromocytoma comprises 11% of all incidental adrenal masses; functional lesions leading to negative consequences are largely dependent on the patient's medical profile and comorbidities. Therefore, primary care physicians should routinely follow up indeterminate incidental adrenal lesions and actively exclude pheochromocytoma or refer to endocrinologist for a definitive work-up in the right clinical settings [20]. Appropriate management includes follow-up with laboratory parameters and imaging such as multiphase contrast-enhanced CT or MRI [20]. For patients with berry aneurysms, certain modifiable risk factors need to be preemptively addressed, including hypertension, smoking, and use of sympathomimetic drugs [19, 20]. Known intracranial aneurysms should be monitored with repeated imaging and intervened upon if there is a high risk for rupture.

## 4. Conclusion

The present case describes an unusual deadly combination of clinicopathologic findings, comprising a functional pheochromocytoma and berry aneurysm ultimately leading to death due to subarachnoid hemorrhage and cardiovascular shock. We highlight the impact of the repeated catecholamine release from a pheochromocytoma, leading to sustained and severe hypertension predisposing to formation of intracranial aneurysm in high risk individuals. It is important to implement preventative medicine and appropriate follow-up measures in patients with incidentally detected adrenal mass or intracranial berry aneurysm, as this could prevent the sequelae of complications such as a ruptured aneurysm or cardiovascular shock, more so in the setting of a functional pheochromocytoma.
